# Patients aged 55 or older undergoing around the knee osteotomy have a higher rate of deep vein thrombosis but not overall early post‐operative complications

**DOI:** 10.1002/jeo2.70023

**Published:** 2024-09-23

**Authors:** Rodrigo Olivieri, José Laso, Tomás Pineda, Pablo Albornoz, Nicolás Starocelsky, Nicolás Franulic, Jaime Ugarte

**Affiliations:** ^1^ Department of Orthopedic Surgery Knee Unit, Hospital del Trabajador ‐ ACHS Santiago Chile; ^2^ Hospital Barros Luco Trudeau Santiago Chile; ^3^ Hospital El Carmen Santiago Chile; ^4^ Universidad Andres Bello, Medicina, Facultad de Medicina Santiago Chile; ^5^ Medical Intern Universidad de Los Andes Chile; ^6^ Hospital Militar de Santiago Santiago Chile

**Keywords:** complications, distal femoral osteotomy, high tibial osteotomy, knee osteotomies

## Abstract

**Purpose:**

Osteotomies around the knee have been established as an effective method for treating varus or valgus malalignment associated with other knee pathologies in young and middle‐aged patients. There is limited literature regarding the risks and complications based on patient age. The purpose of this study is to determine whether age influences as a risk factor for developing intraoperative and early post‐operative complications in patients undergoing osteotomies around the knee.

**Methods:**

A consecutive series of patients over 18 years old who underwent distal femoral osteotomy (DFO) or high tibial osteotomy (HTO) with a minimum follow‐up period of 90 days were included. Demographic characteristics, surgical technique, intraoperative and post‐operative complications up to 90 days were identified. A statistical comparison based on age younger than 55 years or 55 years and older was conducted to determine if patient age acted as a risk factor in the development of complications.

**Results:**

A total of 159 osteotomies were included, of which 129 were HTOs. The average age was 46.16 years, and 118 patients were younger than 55 years. Seven hinge fractures were identified as the only intraoperative complication, while the overall early post‐operative complication rate was 11.32%. The most frequent was deep venous thrombosis (DVT) in 5.66% of cases, followed by deep infection with a total rate of 2.52%. When performing the subgroup analysis by age, we observed a significantly higher rate of DVT in the group aged 55 years and older (*p* 0.036) (odds ratio 3.96 95% confidence interval 1.009–15.533; *p* 0.048); however, no significant differences were observed in the overall complication rate according to the age group of the patients.

**Conclusions:**

This study reveals that in patients undergoing osteotomies around the knee, the most common post‐operative complication was DVT. The rate of DVT was significantly higher in patients aged 55 years and older, although no differences were observed in the overall complication rate according to the patients' age range.

**Level of evidence:**

Level III (retrospective cohort study).

**Abbreviations**
BMI: body mass index
DAIR: debridement, antibiotics, and implant retention
DFO: distal femoral osteotomy
DVT: deep venous thrombosis
HTO: high tibial osteotomy
IQR: interquartile range
PE: pulmonary embolism
SD: standard deviation

## INTRODUCTION

Knee osteotomies have been established as an effective management alternative in the treatment of various pathologies affecting the joint, such as osteoarthritis, congenital and acquired limb axis deformities, isolated chondral lesions, sequelae of meniscectomies, ligamentous instabilities, and consequences derived from fractures and other traumatic pathologies [4, 11, 15, 19, 21, 27, 30, 33, 37].

Depending on the location of the deformity, osteotomies may be performed at the level of the distal femur (DFO), the proximal tibia (HTO), or at dual sites in cases of mixed deformities or with the need for major corrections [36].

Traditionally, these procedures have been reserved for younger or more active patients. In fact, Brinkman et al. [4] define ideal candidates for HTO as patients between 40 and 60 years old. However, there is a significant number of patients over 55 or even 60 years old who undergo DFO or HTO, given their satisfactory medium and long‐term outcomes [22, 40]. With concern to this, there is no consensus in the literature regarding an age limit for performing a knee osteotomy. In the case of HTO, there are studies showing better outcomes in younger patients [16, 18, 20] and others that show no differences [22]. Concerning DFO, the evidence is even scarcer.

In this regard, medical comorbidities [5, 23], tobacco use [23], post‐traumatic etiologies [9], elevated body mass index (BMI) [5], have been identified as potential risk factors for complications in knee osteotomies. However, there is limited evidence regarding age, especially in patients older than 55 years, as an independent risk factor for the development of complications.

The purpose of this study was to characterise the population undergoing DFO or HTO at a single trauma center, describing the reasons for the procedure and identifying post‐operative complications associated with osteotomy and to determine whether age acts as a risk factor for the development of complications. The authors hypothesised that an age of 55 years or older does not act as a risk factor for the development of major complications in the population undergoing knee osteotomies.

## METHODS

### Ethics

All patients provided informed consent for the use of their data for research, and the study was approved by the ethical board in advance.

### Study population

A consecutive series of patients treated at a single academic institution between January 2015 and December 2022 who underwent HTO and/or DFO procedures were identified. Exclusion criteria were age <18 years, concomitant ligamentary procedures and revision osteotomy.

Once eligible patients were identified, the entire sample was split into 2 cohorts based on age: younger than 55 years or 55 years and older.

### Data collection

Clinical and operative data of all eligible patients were reviewed, and demographic, operative, and post‐operative data were obtained.

The following intraoperative and immediate post‐operative complications were assessed: intraoperative hinge fracture, deep vein thrombosis (DVT), pulmonary embolism (PE), death, deep infection, wound infection, wound dehiscence, hemarthrosis, haematoma, knee stiffness, and reoperation.

Short‐term complications study [1]. Early post‐operative complications were defined as those occurring between surgery and the first 90 post‐operative days [3, 23].

Wound infection or dehiscence was identified by antibiotic prescription in response to documented wound concerns or a clear description of gross wound abnormalities. Deep infection was diagnosed based on the presence of a fistula, wound dehiscence that required surgical debridement, two or more positive cultures for the same pathogen, or confirmation through histological examination [29]. Based on our institutional protocol, all patients with clinical suspicion of DVT or PE during this period were evaluated with lower extremity doppler ultrasound or chest computed tomography with contrast, respectively, for diagnostic confirmation. Study data were collected and managed using REDCap (Vanderbilt University).

### Surgical technique

Indications for HTO and DFO included varus or valgus alignment, respectively, in the setting of symptomatic primary or secondary unicompartmental arthritis of the tibiofemoral joint. Uniplanar or biplanar osteotomy was determined at the discretion of the attending surgeon. All patients were treated by one of seven fellowship‐trained knee surgery orthopaedic surgeons.

TomoFix osteotomy plates for distal femur or proximal tibia (Synthes Inc) were used for all procedures. Surgical approaches were not standardised for this study, and the appropriate correction was determined at the discretion of the attending surgeon. Preoperative prophylaxis with cefazolin or vancomycin (in cases of penicillin allergy) according to the institutional protocol was administered in all cases.

### Post‐operative protocols

An institutional thromboembolic event prevention protocol was used in all patients, consisting of daily use of anti‐embolism stockings and weight‐adjusted low molecular weight heparin (dalteparin or enoxaparin) for 21 days.

Formal physical therapy was initiated in the immediate post‐operative period according to the institutional protocol. Full range of knee motion was allowed immediately after surgery. This was complemented with two or three weekly physical therapy sessions starting from the second post‐operative week with the aim of progressive muscle strengthening and improvement of joint range of motion. Regarding weight‐bearing, full weight‐bearing was allowed as tolerated from the first post‐operative day for closing wedge osteotomies, while partial weight‐bearing with two crutches for three weeks followed by progressive weight‐bearing as tolerated was allowed for opening wedge osteotomies or in cases where hinge fracture was suspected. Rehabilitation was maintained until the third month in all patients under follow‐up.

### Statistical analysis

Continuous variables were expressed as means ± standard deviation where appropriate, while the dichotomous variables were expressed as the number and percentage of patients. The Shapiro–Wilk normality test was used to assess the normality of distributions.

The independent samples *t*‐test was used to compare continuous variables between cohorts. Categorical variables were analysed using the chi‐square test. A priori power analysis was not performed because all eligible patients were included in the study.

SPSS (v25; IBM) was used to perform these statistical analyses and statistical significance was set at a p‐value of less than 0.05.

## RESULTS

Of the 162 DFO or HTO surgeries that met the inclusion criteria during the study period, three cases were excluded due to loss of follow‐up.

One hundred and fifty‐nine knees in the same number of patients were included of whom 109 were male (68.55%). The mean age was 46.16 years (median 48; standard deviation [SD] 10.91 years; interquartile range [IQR] [37–55]), and their average body mass index (BMI) was 29.76 kg/m² (median 29.37 kg/m²; SD 4.14 kg/m², IQR [26.64–32.45 kg/m²]), with 69 obese patients (43.4% BMI ≥ 30). Seventy‐three patients (45.91%) had at least one comorbidity, with arterial hypertension being the most frequent in 34 cases (21.38%). In 82 knees (51.57%), the osteotomy was on the right side mean follow‐up was 16.68 months (median 11; SD 15.26; range [3–96 months]) (Table 1).

**Table 1:** Demographic data.

	Total (*n* = 159)	Subgroups		
		<55 yo (*n* = 118)	≥ 55 yo (*n* = 41)	*p*‐Value
Age, mean (SD)	46.16 (10.91)	41.76 (8.96)	58.82 (3.77)	**<0.001**
Male, *n* (%)	109 (68.55%)	83 (70.34%)	26 (63.41%)	0.411
Right, *n* (%)	82 (51.57%)	68 (57.63%)	14 (34.15%)	**0.010**
Medical history, *n* (%)	16 (10.06%)	10 (8.46%)	6 (14.63%)	0.259
Type 2 diabetes	14 (8.81%)	8 (6.78%)	6 (14.63%)	0.126
Immune system disease	2 (1.26%)	2 (1.69%)	0	0.402
Smoker	40 (25.16%)	32 (27.12%)	8 (19.51%)	0.334
BMI, mean (SD)	29.76 (4.14)	29.74 (4.2)	29.82 (3.99)	0.837
Obesity	69 (43.4%)	50 (42.37%)	19 (46.34%)	0.659
Follow‐up, mean (range)	16.68 (3–96)	16.97 (3–96)	15.85 (3–52)	0.619

*Note*: Age expresses in years, follow‐up is expressed in months and obesity is defined as BMI > 30. Bold figures indicate statistical significance (*p* < 0.05).

Abbreviations: BMI, body mass index; SD, standard deviation; yo, years old.

The most frequently described cause for osteotomy was degenerative changes in 107 knees (67.3%), and post‐traumatic osteotomies were performed in 52 cases (32.7%). Osteotomy was performed on the distal femur in 30 cases (18.87%) and proximal tibia in 129 cases (81.13%). Of the femoral osteotomies, 12 were medial (40%) and 18 were lateral (60%). For tibial osteotomies, medial wedges accounted for 125 cases (96.9%) and lateral wedges reached a total of four cases (3.1%) (Table 2). Also, no lateral femoral closing wedge osteotomies were performed.

**Table 2:** Osteotomy characteristics.

	Overall		<55 yo		≥ 55 yo	
	Tibial	Femoral	Tibial	Femoral	Tibial	Femoral
Location, *n* (%)						
Medial opening	105 (81.4%)	4 (13.3%)	74 (77.8%)	4 (17.39%)	31 (91.18%)	0
Medial closing	20 (15.5%)	8 (26.67%)	18 (18.95%)	6 (26.09%	2 (5.88%)	2 (28.57%)
Lateral opening	4 (3.1%)	18 (60%)	3 (3.16%)	13 (71.43%)	1 (2.94%)	5 (71.43%)
Dimensions, mean (SD)						
Medial opening	8.29 (2.96)	7.4 (1.34)	8.35 (3.23)	8.75 (0.95)	8.45 (2.17	0
Medial closing	6.42 (2.62)	7.6 (3.91)	5.3 (2.51)	6 (0)	7 (0)	0
Lateral opening	5.75 (2.06)	7.89 (2.49)	6 (3)	7.69 (2.28)	6 (0)	8 (3.31)

*Note*: Osteotomy dimensions are expressed in millimetres.

Abbreviation: SD, standard deviation.

Regarding the osteotomy itself, opening wedge osteotomies were performed with a mean wedge of 8.13 mm (median 8 mm; SD 2.85 mm, IQR [6–10 mm]), and in the case of closing wedges the closure measured 6.73 mm (median 6 mm; SD 2.94 mm; IQR [4–10 mm]). In either case (opening and closing wedge osteotomies), no significant association was found with the development of post‐operative complications (*p* 0.27). Specifically in tibial osteotomies, medial opening wedge as the most frequent type of osteotomy had a mean wedge base of 8.29 mm (median 8 mm; SD 2.96 mm; IQR [6–10 mm]), and in the case of lateral femoral opening osteotomies, the wedge base measured 7.89 mm (median 7 mm; SD 2.49 mm; IQR [6–10 mm]). Regarding the magnitude of the wedge, the size of opening wedges are not significantly different between patients with and without complications (*p* 0.27). In closing wedge osteotomies, we did not find post‐operative complications.

When examining intraoperative complications, hinge fractures detected during surgery were observed in seven cases (six in the younger group and one in the older patients group) without significant association with age (*p* 0.48). No neurovascular injuries or any other type of intraoperative complications were observed.

When analysing the overall development of complications up to 90 days, we found that patients younger than 55 years old compared with patients 55 years or older did not have significant differences (*p* 0.055). A logistic regression model was fitted to determine the association between age, comorbidities, and smoking status, and the likelihood of developing complications within 90 days. The model failed to demonstrate a significant association between these variables and the outcome of interest (*χ*² = 4.92, *df* = 3, *p* = 0.1781). When assessed by a subgroup of post‐surgical adverse events, the most frequent complication was deep vein thrombosis with four cases in younger patients and five cases in older patients (3.39% vs. 12.2%; *p* 0.036), this complication had a significant association with age group, resulting in an odds ratio (OR) of 3.96 (confidence interval [CI] 1.009–15.533; *p* 0.048). A single case of pulmonary embolism (in the younger patients group), four cases of infection (two in each group), one case of wound dehiscence, severe hemarthrosis and knee stiffness (in the younger patients group), and one case of haematoma managed with serial dressings (in the older group) were also observed, but none of these complications was significantly associated with age group (Table 3).

**Table 3:** Osteotomy complications.

	Total	Subgroups		
		<55 yo	≥ 55 yo	*p*‐Value
Hinge fracture, *n* (%)	7 (4.4%)	6 (5.08%)	1 (2.44%)	0.477
Post‐operative				
Overall, *n* (%)	18 (11.32%)	10 (8.47%)	8 (19.51%)	0.055
DTV, *n* (%)	9 (5.66%)	4 (3.39%)	5 (12.2%)	**0.036**
PE, *n* (%)	1 (0.63%)	1 (0.85%)	0	0.554
Wound dehiscence, *n* (%)	1 (0.63%)	1 (0.85%)	0	0.554
Deep infection, *n* (%)	4 (2.52%)	2 (1.69%)	2 (4.88%)	0.262
Severe hemarthrosis *n* (%)	1 (0.63%)	1 (0.85%)	0	0.554
Haematoma *n* (%)	1 (0.63%)	0	1 (2.44%)	0.089
Knee stiffness *n* (%)	1 (0.63%)	1 (0.85%)	0	0.554

*Note*: Bold figures indicate statistical significance (*p* < 0.05).

Abbreviations: DTV, deep vein thrombosis; PE, pulmonary embolism; yo, years old.

## DISCUSSION

In this study, which presents eight years of experience in knee osteotomies at a single academic and trauma center, the most significant findings are that patients aged 55 and older present a higher risk of DVT in the early post‐operative period and, secondly, that the overall rate of associated complications at 90‐day follow‐up does not correlate with age.

This study found an overall intraoperative complication rate of 4.4%, all of which were hinge fractures, with no observed differences according to patient age. No neurovascular injuries or other types of intraoperative adverse events were observed. Additionally, we identified an overall early post‐operative complication rate of 11.32%, in which no differences were observed according to the age of the subjects.

The most common early post‐operative complication in both groups was DVT, with an overall rate of 5.66%, followed by deep infection requiring surgical debridement in 2.52% of cases.

In a subgroup analysis of post‐operative complications, we found a significant age‐related difference only in the rate of DVT, observed in 12.2% of older patients compared to 3.39% of patients younger than 55 years (*p* = 0.036). For the other early post‐operative adverse events identified in this cohort, namely PE, wound dehiscence not requiring surgical management, deep infection, haematoma, hemarthrosis, or knee stiffness, no significant differences were observed according to age. Due to this, the overall rate of perioperative complications did not show significant differences according to patient demographics.

Both DFO and HTO are widely accepted treatments for managing varus or valgus malalignment [7, 24, 25, 34, 43] in the context of unicompartmental osteoarthritis, instability, or post‐traumatic knee deformity. Although historically age has been defined as a key factor in patient selection [4, 16, 19, 20, 26], other studies have determined that age is not a decisive variable in mid‐term outcomes for these procedures [12, 38, 40, 41].

Regarding the rate of hinge fractures in these types of osteotomies, the literature reports varying rates that reach up to 32% [6, 10, 17, 28, 42]. Berk et al. [3], in their study that includes both DFO and HTO, report an overall rate of 3.7%. Han et al. [13] reports a rate of 12% for non‐displaced hinge fractures and 2.4% for displaced fractures. Song et al. [39] report a 24.2% rate of lateral hinge fractures in HTO, while Ismailidis [17], in his work on indications and complications in DFO, found a 32% rate of fractures. Similarly, Park et al. [32] report an overall fracture rate of 16.1% in their propensity score‐matched analysis study that included only HTO, without finding significant differences by age when comparing patients younger than 55 versus those older than 65 years. In our study, we also did not find differences when adjusting age to 55 years or older, including DFO as well.

Regarding early post‐operative complications, defined as those occurring within 90 days from the surgery date, Kucirek et al. [23], in their extensive study, found adverse event rates of 21.5% and 11.6% for DFO and HTO, respectively, at 3 months post‐surgery. Similarly, Cotter et al. [5] found complication rates of 22.3% and 9.9% for DFO and HTO, with age over 45 years and medical comorbidities being independent risk factors associated with adverse events. In our study, the overall rate of post‐operative complications was 11.95%; however, age influenced the overall rate, and we did not observe differences in medical comorbidities between the two groups.

Although the overall complication rate by age does not show differences, subgroup analysis of complications revealed a higher rate of DVT in the group of patients aged 55 or older (12.2% vs. 3.39%, *p* = 0.036). In the case of this type of complication, the literature varies regarding its incidence. In their comparative study of different knee osteotomy techniques, Onishi et al. [31] found an overall DVT rate of 13.8% when specifically searched for at 7 days postoperatively, whereas Erickson et al. [8] reports a rate of 1.42%. Cotter et al. [5] reported a range from 1.5% for HTO to 2.3% for DFO. In the extensive studies by Ferner et al. and Schenke et al. [9, 35], the reported rates are 0.35% and 0.3%, respectively. This variability in rates suggests potential underdiagnosis due to possible oligosymptomatic presentations. Specifically, regarding age, Lee et al. [26] found no differences in DVT when comparing patients younger than 60 versus those older than 65 years. In our study, we did find an age‐related difference, with a higher incidence in patients over 55 years, despite being subjected to the same thromboprophylaxis protocol. Additionally, we found a relatively high rate of DVT compared to what has been previously reported. This leads us to recommend a more targeted search in this age group and potentially revising the DVT prevention protocols.

**Figure 1:**
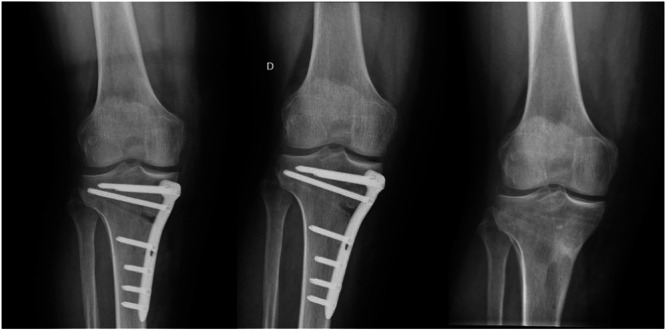
Removal of osteotomy plate in a patient with deep infection diagnosed at 84 days. In the center, a radiograph prior to removal. On the right, a radiograph three months after removal showing consolidated osteotomy.

With respect to other early complications studied, in the case of deep infections, we found an overall rate of 2.52%, with no significant differences by age. This is relatively similar to the 2.8% reported by Hancock et al. [14] in 822 osteotomies. Anagnostakos et al. [2] found a rate of up to 4.7% of deep infections in a specific review of infections in HTO. Berk et al. [3] identified a rate of 4.1%, mainly concentrated in cases following HTO. Cotter et al. [5] reports a cumulative rate of 2.5% in osteotomies around the knee. Unlike our findings, although with a slightly different age distribution (over 65 years), Lee et al. [26] report a higher infection rate in older patients. Nonetheless, the rates reported in the literature remain relatively similar, likely because the symptoms are generally evident, and early treatment can prevent worse complications in the medium and long term. Concerning the management of infections, three of them were diagnosed within 30 days from the surgery date and were managed with debridement, antibiotics, and implant retention (DAIR), and the 4th case was diagnosed 84 days post‐surgery. In this case, which involved an HTO with partial signs of consolidation in a 43‐year‐old patient, the osteotomy plate was removed, and surgical debridement was performed. Staphylococcus epidermidis was isolated and successfully managed with vancomycin and partial weight‐bearing until adequate bone consolidation was achieved during follow‐up (Figure 1).

Regarding other less frequently observed complications, one case of PE unrelated to DVT was identified in the group younger than 55 years. This case involved a 28‐year‐old male smoker, diagnosed at 48 hours and successfully managed initially with dalteparin and subsequently with dabigatran for six months. In this regard, the reported incidences of PE tend to be low. For example, Berk et al. [3] reports 1.2%, while Cotter and Kucirek [5, 23] report overall incidences of 0.1% and 0.94%, respectively. In these cases, the incidences are not significantly different across various studies, which leads us to believe that being a complication with more acute symptoms and potentially devastating consequences, there is a low rate of underdiagnosis.

Additionally, we observed a case of tense hemarthrosis in a 35‐year‐old male patient, which required arthrocentesis on two occasions within 48 hours post‐surgery; a case of haematoma related to the surgical approach in a 56‐year‐old male patient; and a case of knee stiffness in a 37‐year‐old patient who underwent HTO and required arthroscopic fibroarthrolysis eight weeks post‐surgery. In the first case, a fibroarthrolysis was performed during the same surgical procedure due to a history of pre‐existing knee stiffness. Neither of these complications showed a significant difference by age, and all these complications have been described in variable ranges in the literature previously [3, 9, 23, 44].

There are limitations that we can identify in this study. The first is its retrospective nature, which generates inherent biases in the choice of the initial type of surgical intervention and the potential loss of important follow‐up data, primarily limited to the information available in the clinical records and image database, which could lead to underreporting of complications.

Additionally, given the number of surgeons who performed the osteotomies, there are inherent biases in the type and technique of the chosen intervention, which are reflected, for example, in the distribution of techniques used in DFO. Furthermore, due to the study design focused on the patient's age, other potential risk factors such as BMI, previous surgeries, and concomitant procedures were not analysed as independent risk factors but only to assess whether the age groups were comparable in these variables.

Another identified limitation is that, given the study design, only patients over 18 years of age were included, which excludes the paediatric population. Therefore, the results and conclusions are not applicable to patients in that age range.

Among the strengths of the present study, we can highlight the low loss to follow‐up during the study period, with 159 out of 162 surgeries performed (98.1%) included in the final analysis. Another point to emphasise is the uniformity in the hardware used in surgeries, a homogeneous thromboprophylaxis protocol, as well as standardised post‐surgical rehabilitation.

Finally, the analysis for this age range had not been included in previous publications, which allows us, on one hand, to take appropriate precautions when performing surgical interventions in a specific population, and on the other hand, encourages us to confirm these findings in future prospective studies.

## CONCLUSIONS

The present study reveals a higher rate of DVT up to 90 days postoperatively in patients aged 55 or older undergoing DFO or HTO, compared to those under 55. However, in the total rate of intraoperative and post‐operative complications during this same period, we did not observe differences based on the patients' age range. These findings should prompt surgeons to take appropriate precautions for post‐operative management of patients and should be confirmed by future prospective studies.

## AUTHOR CONTRIBUTIONS

We declare that all authors have seen and agreed to the submitted version of the article, and are responsible for it, and that all authors have participated in authorship and the article has not been submitted to any other journal. Rodrigo Olivieri designed the study, coordinated the team, participated in data collection, abstract writing, manuscript drafting, and bibliographic references, and is the corresponding author. José Laso was is responsible for data collection, synthesis and writing of results, and statistical analysis. Tomás Pineda participated in data collection, materials and methods drafting, and table preparation. Pablo Albornoz and Nicolás Starocelsky participated in data collection. Nicolás Franulic participated in data collection and worked on abstract writing and discussion. Jaime Ugarte participated in data collection and development for approval by the ethics committee of our institution.

## CONFLICT OF INTEREST STATEMENT

The authors declare no conflict of interest.

## ETHICS STATEMENT

The authors of this study declare that the following work has received approval from the ethics committee of our hospital (Comité de Ética Clínica del Hospital del Trabajador), and we consent to its publication.

## Data Availability

We also declare that all information and dataset related to the study is available in digital format at https://drive.google.com/drive/u/0/folders/1h3kQcRafm4XOlk683-G3GOgW3CGL3VgK and can be shared upon a reasonable request for academic purposes only.
